# The effects of concurrent bilateral anodal tDCS of primary motor cortex and cerebellum on corticospinal excitability: a randomized, double-blind sham-controlled study

**DOI:** 10.1007/s00429-022-02533-7

**Published:** 2022-08-19

**Authors:** Shabnam Behrangrad, Maryam Zoghi, Dawson Kidgell, Farshad Mansouri, Shapour Jaberzadeh

**Affiliations:** 1 Department of Physiotherapy, School of Primary Health Care, Faculty of Medicine, Nursing and Health Sciences, Monash University, P.O. Box 527, Melbourne, VIC Australia; 2 Department of Rehabilitation, Nutrition and Sport, School of Allied Health, La Trobe University, Bundoora, VIC Australia; 3 Cognitive Neuroscience Laboratory, Department of Physiology, Monash Biomedicine Discovery Institute, Monash University, Melbourne, VIC 3800 Australia

**Keywords:** Noninvasive brain stimulation, Cerebellum, Primary motor cortex, Transcranial magnetic stimulation, Transcranial direct current stimulation

## Abstract

Transcranial direct current stimulation (tDCS) applied to the primary motor cortex (M1), and cerebellum (CB) can change the level of M1 corticospinal excitability (CSE). A randomized double-blinded crossover, the sham-controlled study design was used to investigate the effects of concurrent bilateral anodal tDCS of M1 and CB (concurrent bilateral a-tDCS_M1+CB_) on the CSE. Twenty-one healthy participants were recruited in this study. Each participant received anodal-tDCS (a-tDCS) of 2 mA, 20 min in four pseudo-randomized, counterbalanced sessions, separated by at least 7 days (7.11 days ± 0.65). These sessions were bilateral M1 stimulation (bilateral a-tDCS_M1_), bilateral cerebellar stimulation (bilateral a-tDCS_CB_), concurrent bilateral a-tDCS_M1+CB_, and sham stimulation (bilateral a-tDCS_Sham_). Transcranial magnetic stimulation (TMS) was delivered over the left M1, and motor evoked potentials (MEPs) of a contralateral hand muscle were recorded before and immediately after the intervention to measure CSE changes. Short-interval intracortical inhibition (SICI), intracortical facilitation (ICF), and long interval intracortical inhibition (LICI) were assessed with paired-pulse TMS protocols. Anodal-tDCS significantly increased CSE after concurrent bilateral a-tDCS_M1+CB_ and bilateral a-tDCS_CB_. Interestingly, CSE was decreased after bilateral a-tDCS_M1_. Respective alterations in SICI, LICI, and ICF were seen, including increased SICI and decreased ICF, which indicate the involvement of glutamatergic and GABAergic systems in these effects. These results confirm that the concurrent bilateral a-tDCS_M1+CB_ have a facilitatory effect on CSE, whereas bilateral a-tDCS_M1_ exert some inhibitory effects. Moreover, the effects of the 2 mA, 20 min a-tDCS on the CB were consistent with its effects on the M1.

## Introduction

Transcranial direct current stimulation (tDCS) is a technique of applying a weak direct current (0.5–2 mA) for a relatively long period (usually less than 30 min) to the scalp via two or more surface electrodes. The effects of tDCS are mainly induced by modulation of spontaneous neuronal activity (Nitsche and Paulus 2000; Nitsche et al. 2005; Rossini et al. 2015) and lead to neuroplastic (Karabanov et al. 2015; Cirillo et al. 2017; Huang et al. 2017) and corticospinal excitability (CSE) changes (Nitsche and Paulus 2000; Nitsche et al. 2005; Soekadar et al. 2014; Marquez et al. 2015; Rossini et al. 2015). Over the last two decades, tDCS has gained popularity due to its noninvasive nature, simplicity of use and effects on neuronal activity (Nitsche 2011; Soekadar et al. 2014; Rossini et al. 2015; Vaseghi et al. 2015; Dissanayaka et al. 2017; Morya et al. 2019). Previously it was suggested that anodal tDCS (a-tDCS) acts as a facilitatory technique, increasing spontaneous neuronal activity and CSE, while cathodal tDCS acts as an inhibitory technique, reducing the spontaneous neuronal activity and CSE. However, emerging evidence suggests that the conventional notion about the polarity-dependent effects of tDCS is not always the same (Monte-Silva et al. 2013; Hassanzahraee et al. 2020). This can be explained by metaplastic mechanisms responsible for a-tDCS effects. In addition, these studies suggest that many variables can affect the a-tDCS outcomes, such as duration threshold (Monte-Silva et al. 2013; Hassanzahraee et al. 2020), other than just its polarity. It has been demonstrated the type of plasticity induced can be changed by periodical stimulation, and a specific time window is critical for its induction (Monte-Silva et al. 2013). Therefore, determining the a-tDCS effect is not as straightforward as it seems.

Apart from cortical behaviors, other outcomes were also the interest in tDCS field, such as cognition (Hill et al. 2016; Verissimo et al. 2016; Katsoulaki et al. 2017; Martin et al. 2018), motor learning (Karok and Witney 2013; Ammann et al. 2016; Wiltshire and Watkins 2020; Wang et al. 2021) and postural balance in humans (Kaminski et al. 2013; Ehsani et al. 2017; Saruco et al. 2017, 2018; Manor et al. 2018; Nomura and Kirimoto 2018). However, interestingly, despite the significant functional and structural connection between M1 and cerebellum shown by neuroimaging and transcranial magnetic stimulation (TMS) studies (Bestmann et al. 2004; Jung et al. 2020; Peters et al. 2020; Spampinato et al. 2020), the majority of tDCS studies that targeted these two brain regions utilized single-site stimulation of either of these sites and mainly unilateral to improve related outcomes, such as CSE (Marquez et al. 2015; Dedoncker et al. 2016; Behrangrad et al. 2019), motor control activities, such as balance (Bellebaum and Daum 2007; Kaminski et al. 2016, 2017; Baharlouei et al. 2020), and motor learning (Karok and Witney 2013; Ammann et al. 2016; Wiltshire and Watkins 2020; Wang et al. 2021), etc. Although some found promising results (Steiner et al. 2016; Saruco et al. 2017, 2018; Poortvliet et al. 2018, Baharlouei et al. 2020), some could not find any significant changes (Horvath et al. 2015; Craig and Doumas 2017; Ehsani et al. 2017; Kaminski et al. 2017; Medina and Cason 2017; Pohjola et al. 2017; Steiner et al. 2020; Wiltshire and Watkins 2020; Wang et al. 2021).

This discrepancy can be explained by the structural and functional connectivity of bilateral M1 and bilateral cerebellum that is more noticeable in activities requiring higher degrees of motor control, and activity of both sides of the body, such as postural balance (Bostan et al. 2013; Carrillo et al. 2013; Ishikawa et al. 2016; Spampinato et al. 2020). The cerebellum receives information from different brain areas in the frontal, parietal, temporal, and occipital lobes and funnel them back to M1 through the ventrolateral nuclei of the thalamus (Allen and Tsukahara 1974; Bellebaum and Daum 2007; Bostan et al. 2013). Thus, these cerebello-cortical pathways can be defined as ways of collecting information from widespread areas of the cerebral cortex to help with the smooth execution of each movement through the M1 area (Allen and Tsukahara 1974; Bellebaum and Daum 2007; Bostan et al. 2013). These findings suggest that more exploratory studies are necessary to refine the conventional single-site unilateral a-tDCS technique and introduce a novel tDCS approach that stimulates multiple brain areas concurrently.

Recently, some neuroscience and neurorehabilitation studies suggested an innovative optimization technique, dual-site stimulation, and concluded superiority of this technique over the conventional single-site stimulation (Vaseghi et al. 2015; Hill et al. 2018; Chen et al. 2019; Koshy et al. 2020). Dual-site stimulation is a technique that is theoretically referred to as concurrent stimulation of two functionally related brain sites within the same hemisphere or across opposite hemispheres (Vaseghi et al. 2015; Hill et al. 2018; Chen et al. 2019; Koshy et al. 2020). Furthermore, although many studies are providing significant structural and functional connection between the bilateral M1 and cerebellum in motor control activities, all of the studies have only investigated the effects of unilateral M1 or cerebellum on CSE and cortico-cortical excitability (Dissanayaka et al. 2017; Behrangrad et al. 2019). No study has explored the effect of the dual-site stimulation of bilateral M1 and cerebellum on the CSE and possible neurophysiological mechanisms behind it by assessing cortico-cortical excitability.

Therefore, to establish the limitations of the single-site stimulation and to propose a new framework for future studies, concurrent bilateral dual-site a-tDCS of cerebellum and M1 is utilized in this study. Since the tDCS can modulate the spontaneous firing rate without causing any action potential (Gandiga et al. 2006), it is expected that the excitatory and/or inhibitory postsynaptic potentials induced by dual-site a-tDCS may modulate the neuronal excitability more efficiently than conventional single site a-tDCS. As a proof of concept study, due to the novelty of the proposed approach, this study is investigating how dual-site a-tDCS will affect CSE and the mechanisms underlying these changes. The aims of this study are:

1. To investigate the effects of bilateral a-tDCS of M1 (a-tDCS_M1_) on CSE.
2. To investigate the effects of bilateral a-tDCS of cerebellum (a-tDCS_CB_) on CSE.
3. To investigate the effects of concurrent bilateral a-tDCS of M1 and cerebellum (a-tDCS_M1+CB_) on CSE.
4. To investigate the underlying mechanisms behind the changes on CSE.
5. To compare the effects of bilateral a-tDCS_M1_, bilateral a-tDCS_CB_, concurrent bilateral a-tDCS_M1+CB_ on CSE and cortico-cortical excitability.

## Materials and methods

### Participants

Twenty-one healthy non-smoking volunteers (10 females, 11 males; mean age 23.66 years ± 4.53) were recruited in this study using a simple non-probability sampling method. The sample size was calculated based on the critical effect size generated from a pilot study on eight participants (power of 0.8, α = 0.05, effect size = 0.9). All participants were right-handed, determined by the Edinburgh Handedness Inventory (58.23 ± 8.8) (Oldfield 1971). Participants were included from a pool of young, healthy, non-smoking adults aged 18–35 years.

Exclusion criteria included any history of neurological, rheumatoid, or musculoskeletal disorders, intracranial metal implantation, implanted devices, such as cardiac pacemakers, cochlear implants, medical pumps, or intracardiac lines, consuming medications for any neurological condition (Wassermann 1998; Brunoni et al. 2011). In addition, participants were asked not to consume any alcohol or caffeine 24 h before the experimental sessions and sleep at least 7 h the night before each session. The experimental protocol was performed in accordance with the Declaration of Helsinki and approved by the Human Research Ethics Committee, Monash University, Melbourne, Australia. Informed consent was obtained from all participants included in the study.

### Study design

**Fig. 1:**
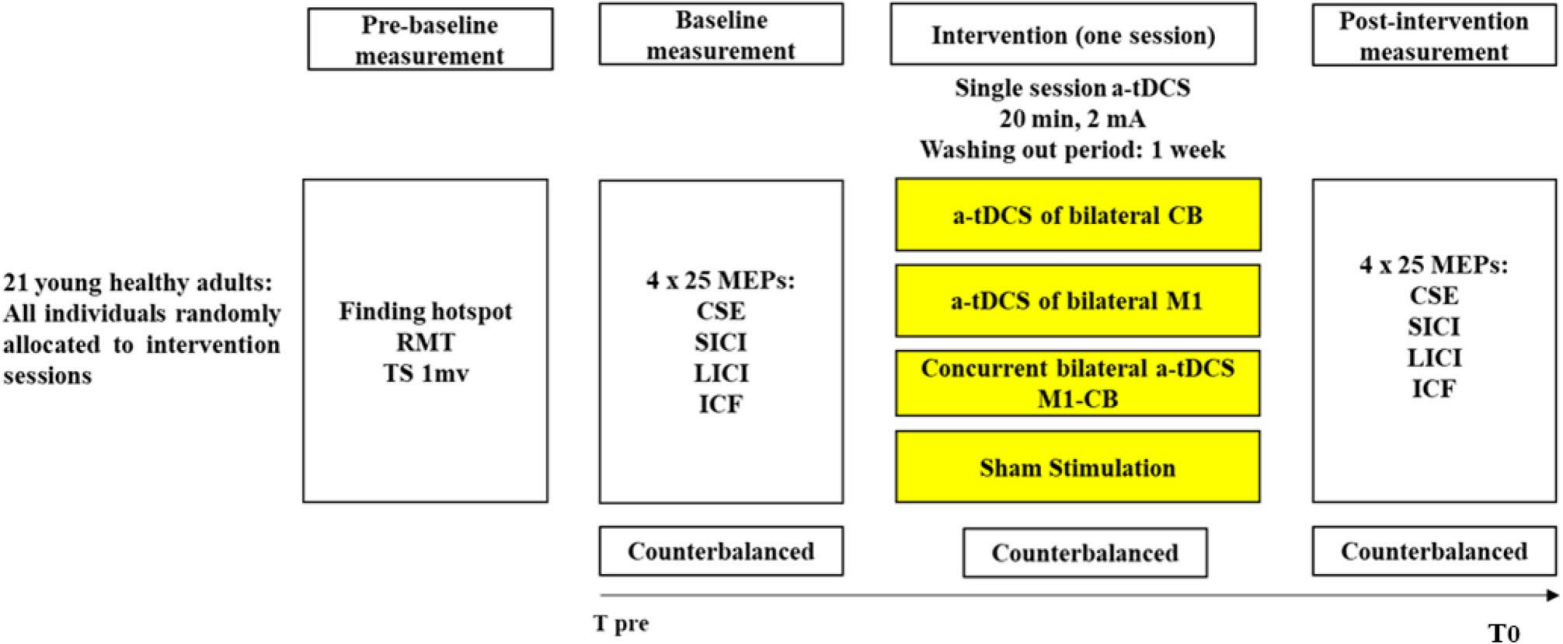
Schematic representation of the experimental procedure for each session. The timeline shows the order of the procedures from left to right. *TMS* transcranial magnetic stimulation, *S* session, *MEPs* motor evoked potentials, *CSE* corticospinal excitability, *ICF* intra-cortical facilitation, *LICI* long intra-cortical inhibition, *SICI* short intra-cortical inhibition, *A-tDCS* anodal–transcranial direct current stimulation, *RMT* resting motor threshold, *TS 1 mV* test stimulus intensity required for peak-to-peak MEP amplitude of approximately 1 mV, *T*_*pre*_ baseline, *T*_0_ Immediately after the intervention

A randomized double-blinded crossover, sham-controlled study design was used in this study. The design involves participation in four experimental conditions (Fig. 1) in a random order: 1. bilateral a-tDCS of M1 (a-tDCS_M1_)_,_ 2. bilateral a-tDCS of cerebellum (a-tDCS_CB_), 3. concurrent bilateral a-tDCS_M1+CB_, and 4. bilateral sham a-tDCS (a-tDCS_Sham_).

All participants attended all four experimental sessions, pseudo-randomly in a counterbalanced manner, separated by at least 7 days (7.11 days ± 0.65) (Boggio et al. 2007). Moreover, to reduce the risk of circadian influences, each participant was examined at the same time of the day for all experimental sessions (Krause and Cohen Kadosh 2014; Li et al. 2015). All participants were blinded to the stimulation conditions and the purpose of this study. The participants were unaware of the allocated session, and the blinding integrity was checked after completion of each session by asking about the nature, active or sham, of the stimulation they had received. Two researchers were involved in the present study, one as the a-tDCS administrator and the other as the assessor of the outcome measures. The administrator that was responsible for delivering a-tDCS interventions was not involved in any data collection or analysis. The assessor, who was responsible for data collection and analysis, was blinded to all experimental conditions and the allocation. All participants received a-tDCS under each of the four different experimental conditions.

## Experimental procedures

### Electromyography

Participants were seated upright in an adjustable chair with the right forearm and the wrist in a pronated and neutral position, resting on a pillow. A standard skin preparation procedure (alcohol cleaning and abrading) of each electrode placement site was done to provide proper surface contact and reduce skin resistance (Gilmore and Meyers 1983). Surface electromyography (EMG) was recorded from the first dorsal interosseous (FDI) at rest using pre-gelled self-adhesive bipolar Ag/AgCl disposable surface electrodes with 2 cm inter-electrode distance (measured from the centers of the electrodes). The location of FDI was determined based on anatomical landmarks, palpation, and voluntary muscle contraction through manually resisted index finger abduction. A ground electrode was placed over the ipsilateral right ulnar styloid process. The EMG raw signals were bandpass filtered (10–500 Hz), amplified by 1000 (1000×), sampled at 1000 Hz. The data were collected on PC software (LabChart™ software, AD Instruments, Australia) via a laboratory analogue–digital interface (PowerLab, AD Instruments, Australia) and stored for later offline analysis.

### Tools for assessment of CSE and intracortical excitability

Single- and paired-pulse magnetic stimuli were delivered by a 70 mm figure of eight magnetic coil (Magstim Company Limited, UK), connected to a MagPro R30 stimulator (Mag Venture, Denmark). The coil was placed over the left M1 for FDI muscle, angled 45° from the midline sagittal plane and tangential to the scalp to ensure that the induced current flowed in a posterior–anterior direction (Rossini and Rossi 1998; Schmidt et al. 2009). The area of stimulation with the largest MEP responses was defined as the "hotspot". This spot was marked on the scalp to maximize the consistency of coil placement throughout the entire experiment. The parameter estimation by sequential testing (PEST) technique was used to determine the resting motor threshold (RMT) (Awiszus 2003; Dissanayaka et al. 2018). The RMT was determined based on the International Federation of Clinical Neurophysiology guidelines (Ziemann et al. 1996; Ilic et al. 2002; Rossini et al. 2015). The RMT was defined as the lowest stimulus intensity to elicit the MEP with a peak-to-peak amplitude of 0.05 mV or more in 3 out of 6 consecutive stimuli in the resting FDI (Devanne et al. 2006; Li et al. 2015). The TMS intensity, defined as a percentage of maximum stimulator output (%MSO), was adjusted to elicit a mean MEP amplitude of about 1 mV peak-to-peak (SI 1 mV) in the resting FDI (Nitsche and Paulus 2000, 2001; Rossini et al. 2015). In this study, the baseline MEP means within the range of 1 mV ± 20% were accepted (Labruna et al. 2016). Both single and paired TMS pulse was utilized based on standard protocols to calculate CSE, Short-interval intracortical inhibition (SICI), intracortical facilitation (ICF), and long interval intracortical inhibition (LICI) (Ziemann et al. 1996; Ilic et al. 2002; Shirota et al. 2010). All TMS assessments were carried out by the same assessor (SHB), who was trained for reliable use of TMS.

### Single-pulse TMS: assessment of corticospinal excitability

Twenty-five single-pulse stimuli with 5-s inter-pulse intervals were delivered, and 25 consecutive elicited MEPs were recorded from the right FDI muscle. The average peak-to-amplitudes of 25 MEPs were calculated before (*T*_pre_) and immediately (*T*_0_) after applying a-tDCS to evaluate the tDCS-induced changes on CSE. The baseline TMS intensity was adjusted to elicit 1 mV peak-to-peak MEP amplitude (Thush study) and is kept constant during post-intervention assessments. It is worth mentioning that no significant differences were found between these two TMS intensities (*p* = 0.4, Cohen’s *d* = − 0.16, CI = − 0.76 to 0.45).

### Paired-pulse paradigm: assessment of intracortical inhibition and facilitation

The same SI 1 mV applied for a single pulse was utilized as a test stimulus for paired pulses, preceded by a suprathreshold or subthreshold conditioning stimulus. Paired-pulse TMS can provide important information about the intracortical inhibitory (SICI and LICI) and excitatory (ICF) neural circuits (Chen et al. 2008; Vucic et al. 2013). In the current study, SICI, LICI, and ICF were measured by paired-pulse TMS (Valls-Sole et al. 1992; Kujirai et al. 1993). In SICI and ICF, a subthreshold conditioning stimulus (80% of RMT) is followed by a suprathreshold test stimulus, SI 1 mV, with an inter-stimulus interval (ISI) of 3 and 10 ms, respectively. In LICI, a suprathreshold conditioning stimulus applied 150 ms prior to the test stimulus (motor threshold of 1mv) (Kujirai et al. 1993). The SICI, LICI, and ICF were calculated using the peak-to-peak amplitude for each elicited MEPs. The size of the conditioned MEP was expressed as a percentage of the unconditioned test MEP to assess the modulations of SICI, LICI and ICF. The test stimulus intensity was adjusted to achieve a baseline MEP of about 1 mV (0.8–1.3 mV) and readjusted the intensity for the paired-pulse recordings after the application of a-tDCS to compensate for the effects of the intervention on the MEP amplitude (Nitsche et al. 2005; Pellegrini et al. 2021a, b).

### Transcranial direct current stimulation

**Fig. 2:**
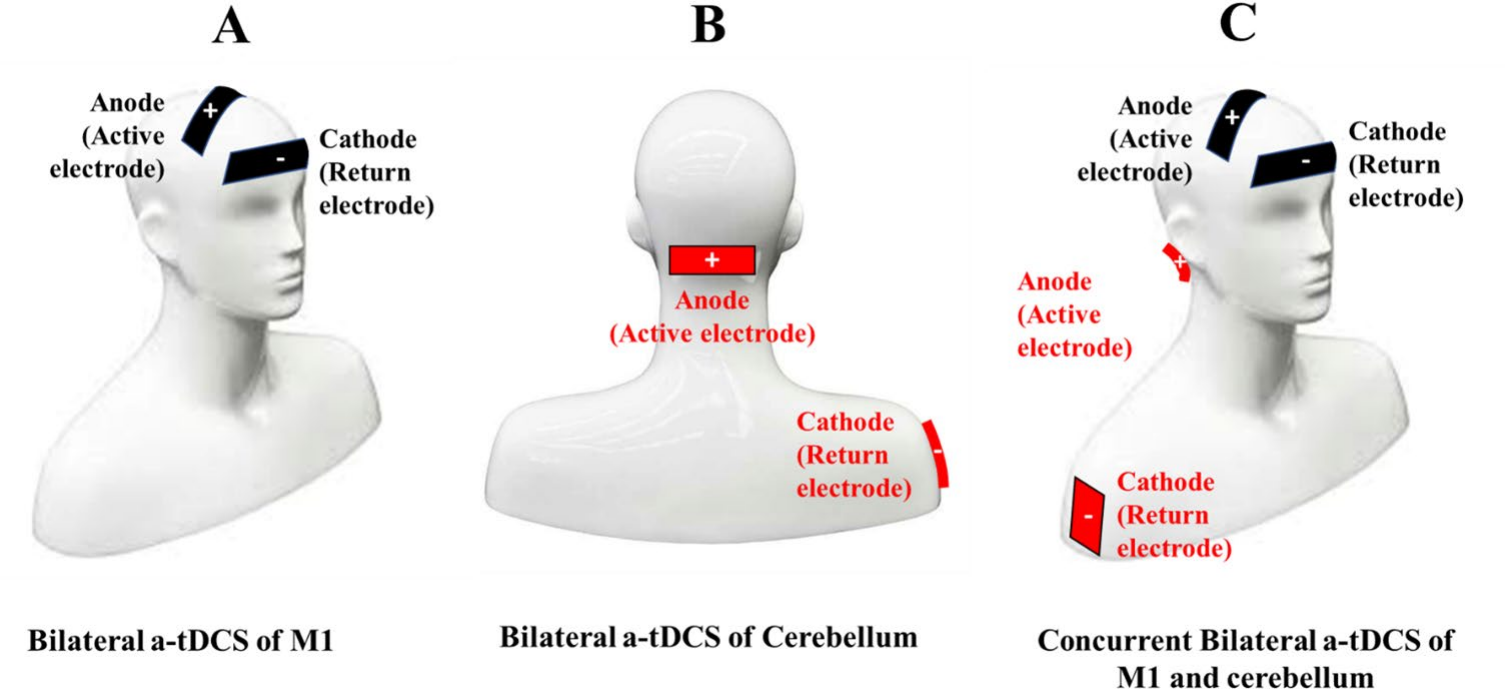
Active electrode placement for **A**: bilateral a-tDCS_CB_, **B**: bilateral a-tDCS_M1_, **C**: concurrent bilateral a-tDCS_M1+CB_

Anodal-tDCS was delivered using a battery-driven direct current stimulator (NeuroConn, Germany) through a pair of saline-soaked surface sponge electrodes. (active, 3 cm × 9 cm, current density: 0.083 mA/cm^2^); return, 5 cm × 7 cm, current density: 0.057 mA/cm^2^) (Fig. 2 shows active electrode placement). For bilateral a-tDCS_M1_, the active electrode (anode) was centered on the Cz, based on the international 10–20 extended EEG system, to cover M1 for bilateral lower extremity, trunk, and upper extremity muscles, and the return electrode (cathode) was placed over the right supraorbital area. The return electrode was deliberately chosen larger to reduce the current density and, therefore, reduce its potential effects on the anterior pole of the brain (Nitsche and Paulus 2000, 2001). For bilateral a-tDCS_CB_, the active electrode (anode) was placed centrally 1 cm below the inion of the occipital bone to cover both the right and left cerebellar hemispheres. The return electrode (cathode) was positioned extracephalic on the right deltoid area (Ferrucci et al. 2015; Ehsani et al. 2017). The landmarks for placing active electrodes were identified by measuring and marking the skull before electrode placement, based on the previous studies (Kaminski et al. 2016; Ehsani et al. 2017; Baharlouei et al. 2020). To facilitate the blinding in this study, the electrode montage used for concurrent bilateral a-tDCS_M1+CB_ was also used for bilateral a-tDCS_M1_, bilateral a-tDCS_CB,_ and bilateral a-tDCS_sham_. Both M1 and CB channels were turned on for bilateral stimulation, while for single-site bilateral a-tDCSM1 and concurrent bilateral a-tDCS_M1+CB,_ only one of the channels was turned on. For a-tDCS_sham,_ both or one of the channels were turned on but for only 30 s.

The electrodes were fixed with two horizontal and perpendicular elastic straps. Two pairs of electrodes for both M1 and CB stimulation were applied over the designated positions in all experimental conditions. Each pair of electrodes were connected to a separate a-tDCS device. In each experimental session, depends on the experimental condition, one or both devices were turned on. The current intensity was set at 2 mA, and the duration of stimulation was 20 min with a 15 s fade-in at the start and 15 s fade-out at the ends of stimulation to minimize the abrupt changes in current intensity and, therefore, discomfort. In the sham experiment, the a-tDCS was turned off after 30 s (Gandiga et al. 2006) (Fig. 1).

### Assessment of the side effects

All participants were asked to answer a questionnaire concerning the side or adverse effects of stimulation in all four experimental conditions at 0–5 min, 6–10 min, 11–15 min, and 16–20 min of the stimulation time. The questionnaire included rating scales for common side effects, such as itching, tingling, burning sensation, or any other side effects under the electrodes (Boggio et al. 2007; George and Aston-Jones 2010; Brunoni et al. 2011). All participants were asked to rate the intensity of each item during and after stimulation based on a numerical analog scale, with 0 representing "no sensation" and 10 representing "the worst sensation imaginable". The items included numbness, itching, burning sensation, pain, fatigue, and headache. In addition, at the end of each experiment, participants were requested to indicate the nature of the stimulation they received (active or sham) by choosing' Yes', 'No', or 'cannot say' as the answer.

### Data analysis

Peak-to-peak amplitudes of 25 single-pulse MEPs were automatically calculated online for each time point of measurement, using a custom-designed macro in Powerlab data recording and analysis 8/30 software (ADInstruments, Australia).. The size of the conditioned MEP was calculated as a percentage of the unconditioned test MEPs to calculate SICI, LICI, and ICF. The data with no knowledge of experimental conditions were blindly analyzed by SPSS version 22 (IBM Corp., Armonk, NY, USA). A one-way repeated measure ANOVA (RM-ANOVA) on baseline values in different experimental sessions for all dependent variables (RMT, SI 1 mV, CSE, SICI, LICI, and ICF) was carried to rule out the carry-over effects of experimental conditions. The normal distribution of data for each outcome measure was examined by the Shapiro–Wilk test, and all variables were normally distributed. The effects of two independent variables, i.e., "the experimental conditions" with four levels (M1, cerebellum, dual-site, and sham stimulations) and "time" with two levels (*T*_pre_, *T*_0_), on CSE, SICI, LICI, and ICF, were assessed through a two-way repeated measures ANOVA. Mauchly's test was carried out to determine the validity of the sphericity assumption for repeated measures ANOVA. The Greenhouse–Geisser corrected significance values were used when sphericity was lacking (Meyers et al. 2006). When ANOVA showed significant results (*p* < 0.05), post-hoc comparisons were performed using the Bonferroni correction.

Furthermore, to determine whether participants were effectively blinded to the stimulation condition (active or sham), participants were asked if they could differentiate between stimulation they received after completing each experiment. The Pearson's chi-square test was carried out on rating scales recorded by questionnaire. Moreover, for side effect analysis, a one-way RM-ANOVA was carried out on the mean values of the rating scale recorded to evaluate any significant differences between the participants' feelings during active and sham conditions. The critical level of significance was set to *p* < 0.05. All results in tables and figures are displayed as means ± standard error measurements (SEM). However, the participant's sensation scores during experimental conditions were reported as means ± standard deviation (SD). In addition, based on the null hypothesis statistical test (significance tests and hypothesis tests), significant statistical testing is not enough to rely on, as it provides information about the existence of the effects (Herbert 2019). Therefore, Cohen's *d* effect size (Cohen 1992; Greenfield et al. 1997; Hickey et al. 2018) was calculated to estimate the effect size of the included studies. According to the thresholds explained by Cohen, the effect size magnitude was interpreted as small (*d* = 0.20), moderate (*d *= 0.50), and large (*d* = 0.80) (Cohen 1992). In this study, the *p* value, followed by the Cohen's *d* effect size and 95% confidence interval (95% CI), is reported in the results.

## Results

All 21 healthy participants completed all experimental sessions. The Shapiro–Wilk test showed normality in all data sets. The results of the one-way RM-ANOVA showed no significant difference in baseline values for RMT, SI 1mv, and MEPs (CSE, SICI, LICI, and ICF) at all experimental conditions (Table 1).

**Table 1:** Baseline TMS measurements

Baseline measurements	Bilateral a-tdcs_M1_	Bilateral a-tdcs_CB_	Concurrent bilateral a-tdcs_M1+CB_	Bilateral a-tdcs_sham_	*df*	*F* value	*p* value
Application of a-tDCS in different experimental conditions							
MT 1 mV (%)					3		
CSE (mV)	1.44 ± 0.45	1.32 ± 0.28	1.13 ± 0.28	1.32 ± 0.31	3	2.51	0.064
SICI (%)	55.41 ± 50.15	43.63 ± 46.05	42.54 ± 32.35	67.28 ± 31.09	3	1.83	0.147
ICF (%)	122.77 ± 34.95	136.17 ± 41.73	139.66 ± 63.55	134.65 ± 67.78	3	0.736	0.534
LICI (%)	39.23 ± 29.4	36.21 ± 30.01	31.92 ± 18.48	34.76 ± 22.54	3	0.514	0.584

Means ± standard deviation (SD)

*MT 1 mV* stimulus intensity required for induction of 1 mV MEP, *CSE* corticospinal excitability, *SICI* short latency intracortical inhibition (% conditioned MEP/Test MEP), *ICF* intracortical facilitation (% conditioned MEP/Test MEP), *LICI* long latency intracortical inhibition (% conditioned MEP/Test MEP)

### The effects of bilateral a-tDCS on CSE

**Fig. 3:**
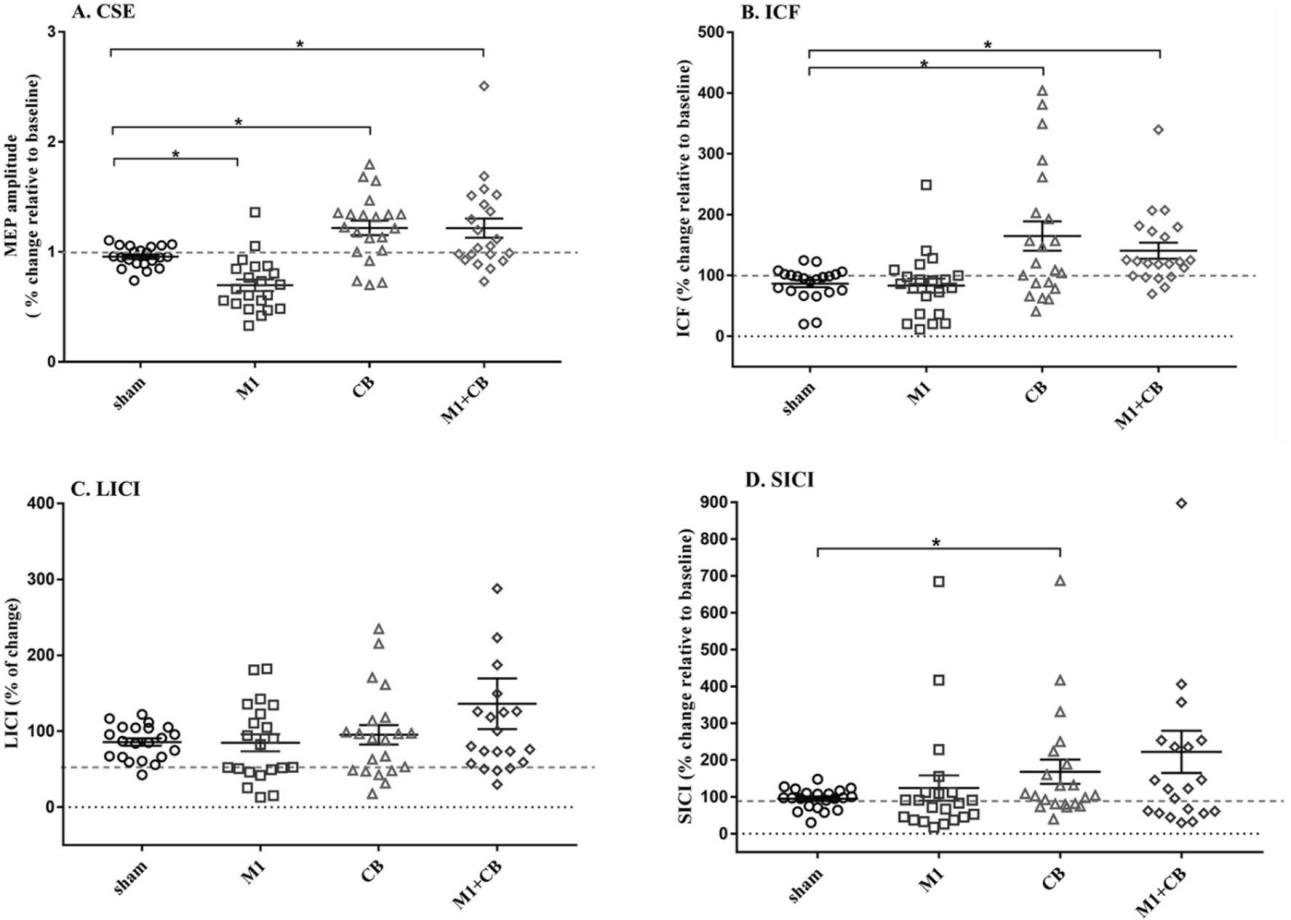
Comparison of the effects of bilateral a-tDCS _M1_, concurrent bilateral a-tDCS_M1+CB_, bilateral a-tDCS_CB_, with bilateral a-tDCS_sham_ on the percentage of changes of the peak-to-peak amplitude of MEPs (**A**), ICF (conditioned MEP/test MEP × 100) (**B**), LICI (conditioned MEP/Test MEP × 100) (**C**), and SICI (conditioned MEP/test MEP × 100) (**D**). The (*) shows significant differences, *p* < 0.05. All of the data are normalized, and ratios of the percentage changes have been mentioned. Each dot represents one participant. Data are reported as mean ± SEM. Lines show the means. Error bars indicate SEM

The two-way RM-ANOVA indicated significant main effects of the experimental conditions (*F* = 4.18, *p* = 0.009) and interaction of condition and time (*F* = 8.927, *p* < 0.0001). However, the results did not reveal any significant main effect of time (*F* = 0.007, *p* = 0.933). Figure 2 summarizes the CSE changes in all participants in all four experimental conditions. The post-hoc comparisons with Bonferroni corrections revealed significant difference in MEP amplitude between bilateral a-tDCS_M1_ and concurrent bilateral a-tDCS_M1+CB_ (*p* = 0.002, Cohen’s *d* = 1.67, 95% CI 0.94–2.34), and bilateral a-tDCS_M1_ and bilateral a-tDCS_CB_ (*p* < 0.0001, Cohen’s *d* = 1.6, 95% CI 0.88–2.26) (Fig. 3).

**Fig. 4:**
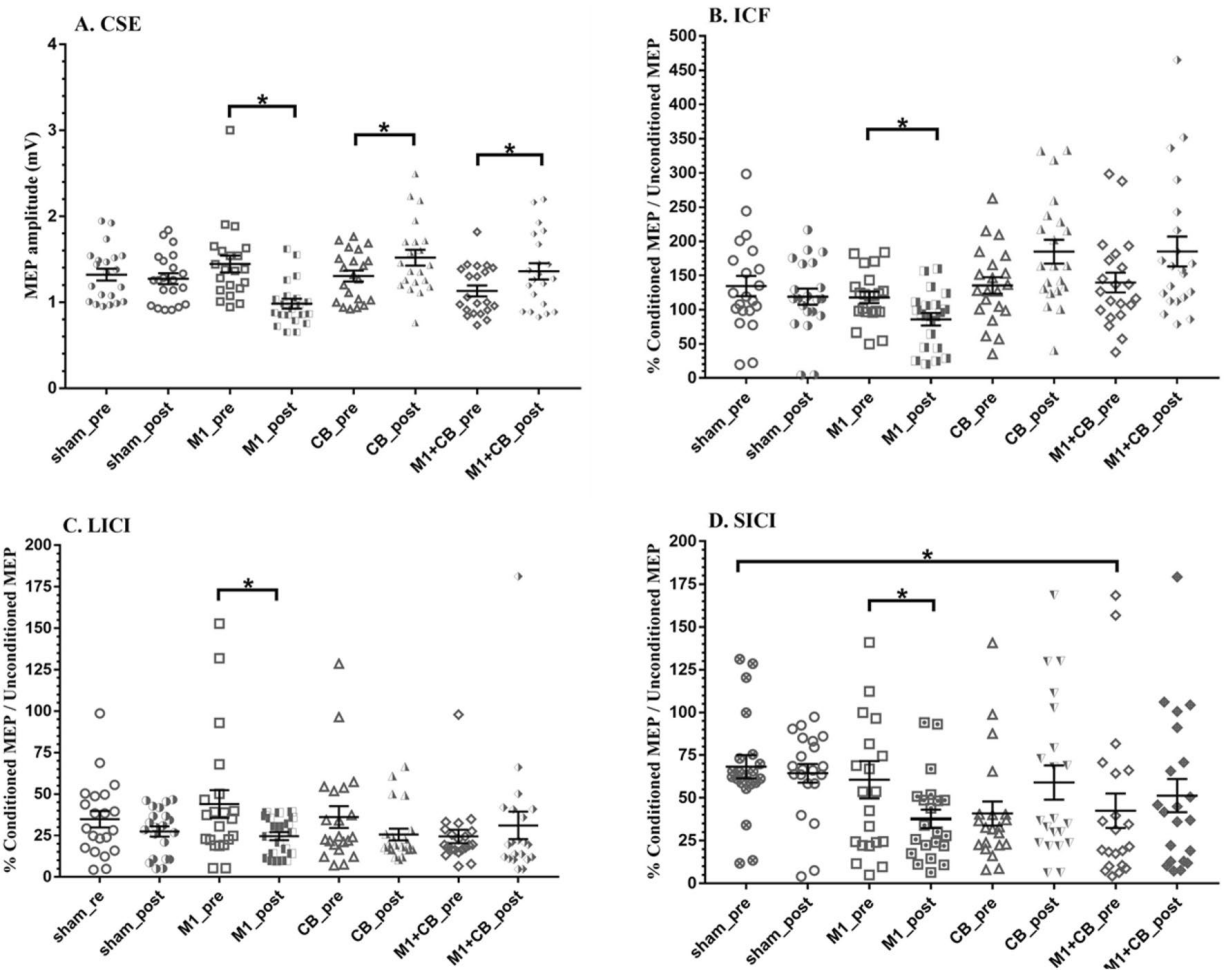
Effects of a-tDCS on the peak-to-peak amplitude of MEPs (**A**), ICF (**B**), LICI (**C**) and SICI (**D**) with bilateral a-tDCS_M1_, concurrent bilateral a-tDCS_M1+CB_, bilateral a-tDCS_CB_, and bilateral a-tDCS_sham_. The (*) shows significant differences, *p* < 0.05. Each dot represents one participant. Data are reported as mean ± SEM. Lines show the means. Error bars show SEM

Similarly, the comparison of bilateral a-tDCS_sham_ with a-tDCS_M1_ (*p* = 0.033, Cohen’s *d* = 1.29, 95% CI 0.6–1.92), a-tDCS_CB_ (*p* = 0.049, Cohen’s *d* = − 1.24, 95% CI − 1.87 to − 0.56), respectively, and concurrent bilateral a-tDCS_M1+CB_ (*p* = 0.02, Cohen’s *d* = 0.92, 95% CI 0.27–1.54). In addition, comparing the peak-to-peak MEP amplitudes of *T*_pre_ and *T*_0_ provided significant decrease following bilateral a-tDCS_M1_ (*p* = 0.01, Cohen’s *d* = − 1.03, 95% CI − 1.66 to − 0.37). Moreover, significant increase were found following bilateral a-tDCS_CB_ (*p* = 0.037, Cohen’s *d* = 0.47, 95% CI − 1.07 to 0.15) and concurrent bilateral a-tDCS_M1+CB_ (*p* = 0.048, Cohen’s *d* = 0.26, 95% CI − 0.36 to 0.86). In addition, comparison of the peak-to-peak MEP amplitudes of *T*_pre_ and *T*_0_ in sham stimulation didn’t show any significant changes (*p* = 0.35, Cohen’s *d* = − 0.07, 95% CI − 0.67 to 0.54). Figure 4 summarizes the CSE changes in all participants in each experimental condition.

### The effects of bilateral a-tDCS on SICI

The RM-ANOVA showed a significant effect of condition and time interaction (*F* = 4.789, *p* = 0.027). However, there was no significant main effect of condition (*F* = 3.928, *p* = 0.103) or time (*F* = 0.011, *p* = 0.916). A significant decrease was seen in the SICI level after bilateral a-tDCS_M1_ application (*p* = 0.041, Cohen’s *d* = − 0.47, 95% CI − 1.07 to 0.15) compared to the respective baseline values (Fig. 4). However, no significant changes were seen comparing *T*_pre_ and *T*_0_ bilateral a-tDCS_CB_ (*p* = 0.532, Cohen’s *d* = 0.45, 95% CI − 0.17 to 1.05), and concurrent bilateral a-tDCS_M1+CB_ (*p* = 0.196, Cohen’s *d* = − 0.14, 95% CI − 0.74 to 0.47). Moreover, significant changes found between bilateral a-tDCS_CB_ and bilateral a-tDCS_Sham_ (*p* = 0.034, Cohen’s *d* = − 0.38, 95% CI − 0.98 to 0.24) (Fig. 3). However, no significant differences were found between the sham and other experimental conditions (*p* a-tDCS_M1_ = 0.93, Cohen’s *d* a-tDCS_M1_ = − 0.5, 95% CI − 1.07 to 0.15) (*p* a-tDCS_M1+CB_ = 0.05, Cohen’s d a-tDCS_M1+CB_ = 0.19, 95% CI − 0.42 to 0.79) (Fig. 3).

### The effects of bilateral a-tDCS on LICI

The RM-ANOVA revealed a significant interaction of stimulation (*F* = 7.679, *p* = 0.001) and interaction of condition and time (*F* = 6.192, *p* = 0.005). Whereas, there was no significant main effect of time (*F* = 1.671, *p* = 0.211). Significant increases were seen in the LICI after bilateral a-tDCS_M1_ compared to respective baseline values (*p* = 0.031, Cohen’s *d* = − 0.66, 95% CI − 1.26 to − 0.02) (Fig. 4). However, no significant changes were seen in the after bilateral a-tDCS_CB_ (*p* = 0.383, Cohen’s *d* = − 0.47, 95% CI − 1.08 to 0.15), concurrent bilateral a-tDCS_M1+CB_ (*p* = 0.166, Cohen’s *d* = 0.19, 95% CI − 0. 42 to 0.79), and a-tDCS_Sham_ (*p* = 0.509, Cohen’s *d* = − 0.24, 95% CI − 0.84 to 0.38) as compared to the respective baseline values (Fig. 4). Moreover, no significant changes were seen between experimental conditions compared with a-tDCS_Sham_ conditions (*p* a-tDCS_M1_ = 0.465, Cohen’s *d* a-tDCS_M1_ = − 0.33, 95% CI − 0.93 to 0.28) (*p* a-tDCS_CB_ = 0.578, Cohen’s *d* a-tDCS_CB_ = 0.26, 95% CI − 0.35 to 0.86) (*p* a-tDCS_M1+CB_ = 0.342, Cohen’s *d* a-tDCS_M1+CB_ = 0.41, 95% CI − 0.21 to 1.02) (Fig. 3).

### The effects of bilateral a-tDCS on ICF

The results of RM-ANOVA provided a significant main effect of ‘conditions’ on ICF (*F* = 7.679, *p* < 0.001), and a significant interaction of condition and time (*F* = 6.192, *p* = 0.005). However, there was no significant main effect of ‘time’ for ICF (*F* = 1.671, *p* = 0.211). Pairwise comparisons showed that ICF decreased significantly following bilateral a-tDCS_M1_ (*p* = 0.01, Cohen’s *d* = − 0.96, 95% CI − 1.57 to − 0.3) compared to the baseline (Fig. 4). However, no significant differences were found after bilateral a-tDCS_CB_ (*p* = 0.13, Cohen’s *d* = 0.7, 95% CI 0.06–1.3) or concurrent bilateral a-tDCS_M1+CB_ (*p* = 0.09, Cohen’s *d* = 0.53, 95% CI − 0.09 to 1.14). In addition, a significant decrease were found in the ICF level after bilateral a-tDCS_CB_ (*p* ˂ 0.005, Cohen’s *d* = 1, 95% CI 0.34–1.62) and concurrent bilateral a-tDCS_M1+CB_ (*p* = 0.013, Cohen’s *d* = 1.04, 95% CI 0.38–1.66) compared with bilateral a-tDCS_Sham_ (Fig. 3).

### Safety and side effects of a-tDCS

Participants' experiences and side effects were recorded at 0–5 min, 6–10 min, 11–15 min, and 16–20 min of stimulation. The means ± SEM of participant's reported side effects for all experimental sessions is summarized in Table 2. No side effects were found after a-tDCS other than light tingling sensations and itching under the electrodes during stimulation reported by some of the participants in all experimental conditions. Itching and tingling under the anode electrode were the most commonly reported side effects. Based on the result, the most severe tingling (mean value of 4.9 ± 0.15) at the beginning of dual-site stimulation and itching (mean value of 4.8 ± 0.45) at the beginning of M1 stimulation were recorded under the anode electrode. No adverse effects of a-tDCS such as burning sensations, headaches, or pain were detected during or after the single or concurrent bilateral dual-site stimulations.

**Table 2:** Participant’s sensation scores (means ± SD) during experimental conditions

Side effects		Anode (active electrode)				Cathode (return electrode)			
		Bilateral a-tDCS_sham_	Bilateral a-tDCS_M1_	Bilateral a-tDCS_CB_	Concurrent bilateral a-tDCS_M1+CB_	Bilateral a-tDCS_sham_	Bilateral a-tDCS_M1_	Bilateral a-tDCS_CB_	Concurrent bilateral a-tDCS_M1+CB_
Tingling sensation	0–5 min	4.8 ± 0.32	4.6 ± 0.4	4.3 ± 0.22	4.9 ± 0.15	2 ± 0.23	1.7 ± 0.1	1.6 ± 0.14	2.1 ± 0.13
	6–10 min	4.1 ± 0.18	3.8 ± 0.15	3.4 ± 0.31	4.5 ± 0.10	2.1 ± 0.14	1.1 ± 0.18	1.0 ± 0.07	0.7 ± 0.10
	11–15 min	3.1 ± 0.23	3.8 ± 0.15	3.4 ± 0.31	3.6 ± 0.13	1.4 ± 0.10	1.2 ± 0.2	1.0 ± 0.07	0.7 ± 0.10
	16–20 min	1.1 ± 0.35	0.8 ± 0.118	0.9 ± 0.1	1.2 ± 0.17	0.5 ± 0.08	0.6 ± 0.16	0.6 ± 0.07	0.7 ± 0.08
Itching sensation	0–5 min	3.1 ± 0.21	4.8 ± 0.45	4.6 ± 0.37	5.1 ± 0.42	1.1 ± 0.09	1.2 ± 0.15	1.3 ± 0.09	1.8 ± 0.11
	6–10 min	1.1 ± 0.14 1.2 ± 0.15	2.6 ± 0.03	2.5 ± 0.06	2.8 ± 0.08	0.6 ± 0.25	0.8 ± 0.12	1.1 ± 0.12	0.6 ± 0.17
	11–15 min	1.2 ± 0.23	2.2 ± 0.03	1.7 ± 0.1	2.3 ± 0.34	0.4 ± 0.06	0.3 ± 0.9	0.3 ± 0.12	0.6 ± 0.09
	16–20 min	1.7 ± 0.15	1.3 ± 0.11	1.8 ± 0.22	1.9 ± 0.16	0.4 ± 0.08	0.5 ± 0.09	0.5 ± 0.11	0.6 ± 0.12

Furthermore, Pearson's chi-square test was conducted to evaluate the success of blinding. The results showed no significant differences between the active and sham conditions (*p*_M1_ = 0.68, *p*_CB_ = 0.75, *p*_M1+CB_ = 0.69), demonstrating that participants could not differentiate between the active and sham stimulations. The majority of participants were properly blinded, and 75% of participants (excluding 'cannot say' responders) could not correctly guess the nature of the a-tDCS condition they had been received, which indicates that the blinding of the participants was successful in this study.

## Discussion

This study compared the effects of a single session of concurrent bilateral a-tDCS_M1+CB_ with bilateral a-tDCS_M1_ or bilateral a-tDCS_CB_ on the CSE in twenty-one healthy young participants. The mechanisms behind the changes in CSE were also investigated using SICI, LICI, and ICF. The results indicate, all three stimulation conditions induced significant changes in CSE compared to its baseline (comparing *T*_pre_ and *T*_0_), while the bilateral a-tDCS_M1_ cause a significant decrease in the CSE level. In addition, the large effect sizes comparing the CSE after bilateral a-tDCS_M1_ with sham stimulation suggest a clinically meaningful reduction in the CSE level after bilateral a-tDCS_M1_ compared to the sham stimulation. In addition, the results show a large effect size when comparing bilateral a-tDCS_CB_ and concurrent bilateral a-tDCS_M1+CB_ to the sham stimulation, which indicates that these stimulation protocols are clinically meaningful for enhancing the CSE. Moreover, a lack of significant differences between the baseline assessments in different experimental conditions suggests that the length of the washing-out period to avoid the carry-over effect among the stimulation conditions was adequate. The results also showed that both single-site and dual-site applications of a-tDCS were well tolerated, and the blinding integrity was successfully achieved.

### The effects of bilateral a-tDCS_M1_ on CSE

The results indicate a reduction in CSE level after bilateral a-tDCS_M1_. This study also demonstrated that the SICI was enhanced and the ICF reduced after bilateral a-tDCS_M1_, which explains this modulation. These changes suggesting the effects of bilateral a-tDCS_M1_ on CSE is inhibitory. Therefore, it may decrease the excitability of intracortical inhibitory interneurons and consequently increase the SICI level and decrease ICF level. Interestingly, these findings suggest that the 20 min of 2 mA a-tDCS of the M1 is shifting CSE from mechanisms associated with long term potentiation (LTP) plasticity, which was conventionally expected (Nitsche and Paulus 2000; Nitsche et al. 2005), to mechanisms associated with long term depression (LTD)-like plasticity. Although no study used bilateral a-tDCS_M1_ similar to this study to compare, the results of this study are in line with findings of other studies that used unilateral a- tDCS_M1_ conducted on healthy humans, which reported that the effects of a-tDCS are not linear and facilitatory, and depends on their parameters it can even decrease M1 CSE (Monte-Silva et al. 2009; Hassanzahraee et al. 2020).

The possible mechanisms behind these brain excitability alterations can be explained by the glutamatergic plasticity involving NMDA receptors (Liebetanz et al. 2002; Nitsche et al. 2003, 2004). Primarily, it has been shown that the activation of the NMDA receptors results in cellular calcium influx and thus affects synaptic plasticity. Based on the activation level of NMDA receptors, the leading effects on calcium influx and synaptic plasticity would be different. It has been provided that low calcium level results in LTD, high calcium increases induce LTP, and calcium overflow again results in LTD (Mosayebi Samani et al. 2019, 2020). Thus, it is speculated that 2 mA tDCS on M1 resulted in LTD-like plasticity due to calcium overflow. This calcium overflow may lead to counteracting potassium channel activation, limiting calcium influx (Yasuda et al. 2003; Misonou et al. 2004; Segal and Korkotian 2016) seems to convert effects. However, these explained mechanisms are speculative and should be explored and confirmed by future pharmacological studies.

### The effects of bilateral a-tDCS_CB_ on CSE

The results indicate an increase in the level of CSE after bilateral a-tDCS_CB_. Regarding cerebellar stimulation, this study showed the 2 mA, 20 min of a-tDCS is acting as an inhibitory technique on the CB, which inhibits the inhibitory effects of CB on M1 and facilitates the M1 increase in the level of CSE. In addition, this finding was supported by the rise in the level of ICF after bilateral a-tDCS_CB_ compared to sham stimulation. These effects can be explained based on the physiology of the CB and M1 connections. Anatomically one of the main cerebellar efferent pathways to the M1 is called the cerebello-thalamo-cortical pathway (Holdefer et al. 2000; Grimaldi et al. 2014), arising from the cerebellar Purkinje cells to the M1 through the dentate nucleus and thalamus (Holdefer et al. 2000; Habas et al. 2009; Grimaldi et al. 2014; Tremblay et al. 2016; D'Angelo 2018). According to the inhibitory action of Purkinje cells, activation of Purkinje cells inhibits the dentate nucleus; the inhibited dentate cells send less excitatory stimuli to the ventrolateral thalamus and subsequently to the M1. Therefore, by inhibiting the CB, the Purkinje cells will be inhibited, and the inhibition of the dentate nucleus will be decreased. This means the dentate nucleus will send more excitatory stimuli to the thalamus and M1, and consequently, an increase in the level of CSE will be seen. Some recent reviews showed a lack of enough information surrounding the behavior of cerebellar a-tDCS on M1 excitability (Fernandez et al. 2018; Behrangrad et al. 2019). However, few studies evaluated the effects of the bilateral 2 mA, 20 min a-tDCS of the CB, and did not find any significant difference in the level of CSE (Galea et al. 2009; Bradnam et al. 2015; Craig and Doumas 2017; Summers et al. 2018; Ehrangrad et al. 2019).

One of the reasons can be the difference in electrode montage. According to the latest finding, it seems that placing the active electrode on the inion or a maximum of 1.5 cm below the inion increases the chance of stimulating the posterior and inferior parts of the CB (i.e., lobules VI–IIX) and hence significant effects after cerebellar a-tDCS (Behrangrad et al. 2019; Behrangrad 2021). The other possible reason seems to be the low number of participants included in these studies. The findings of this study shed light on the effects of cerebellar a-tDCS on the CSE. It is believed that interpreting the effects of the cerebellar a-tDCS on the M1 is not as simple as it seems, and more research is needed to be done to find the effects of cerebellar a-tDCS on the CSE and its behavior (Behrangrad et al. 2019).

### The effects of concurrent bilateral a-tDCS_M1+CB_ on CSE

According to the results, a significant increase, with a large effect size, was seen in CSE of the concurrent bilateral a-tDCS_M1+CB_ compared to bilateral a-tDC_Sham_. In addition, a significant increase in the CSE is seen after the concurrent bilateral a-tDCS_M1+CB_ compared to baseline. This CSE increase can be explained by the significant increase found in the level of ICF of the concurrent bilateral a-tDCS_M1+CB_ compared to bilateral a-tDC_Sham_. It is speculated that the stimulation of M1 and CB reduces the GABAergic intracortical inhibition, which can be interpreted as a decrease in corticospinal neuron inhibition, causing an increased level of CSE. Furthermore, it is speculated that the concurrent bilateral a-tDCS_M1+CB_ may shift the cortical excitability to LTP-like plasticity, which can be explained by increasing the activity of NMDA receptors and subsequent increase in calcium influx (Mosayebi Samani et al. 2019, 2020). This study is the first to investigate the effects of concurrent bilateral a-tDCS_M1+CB_, so further research is needed to support or disprove the results of this study.

### Limitations of the study

The findings in this study should be interpreted considering its limitation. In this study, the effects of each stimulation condition were only assessed immediately after the interventions. This may limit our understanding of possible delayed plasticity changes. In addition, this study was carried out on young, healthy participants (between 18 and 40 years old); thus, the results may not be generalized to older adults or patients with pathological conditions that may not respond similarly to these techniques. In addition, because this study did not investigate gender as a variable, the results of this study are not gender-specific. In addition, although this study, as a proof-of-concept study, tried to investigate the underlying mechanisms behind the CSE changes of the stimulation conditions, this study could not completely disentangle whether CSE changes seen in dual-site stimulation is due to tDCS profound effect on the cerebellum or M1. Consequently, this could be an aim for future studies to have deeper understanding on the mechanisms behind the results found in this study.

### Suggestions for future studies

Longer follow-ups are needed to evaluate the lasting effects of this stimulation technique. These data can be valuable for future studies investigating an optimal approach to improve the CSE of the M1. It is also essential to find out the effects of gender on the results of this stimulation technique in future research, to have more accurate and gender-specific results. It would be important to examine the effects of this a-tDCS technique on older adults and patients with different pathological conditions in future studies. In addition, future studies are necessary to investigate the behavioral outcome measures along with the neurophysiological changes.

## Conclusion

The results of this study indicate that concurrent bilateral a-tDCS_M1+CB_ and bilateral a-tDCS_CB_ able to enhance M1 CSE and induce LTP-like plasticity. However, the results showed that the bilateral a-tDCS_M1_ stimulation might act as an inhibitory intervention rather than facilitatory, inducing LTD-like plasticity. In addition, this study showed that the effects of the a-tDCS for induction of increased CSE are not facilitatory all the time. Therefore, further investigations on the metaplastic mechanisms of this new approach are essential to produce efficient therapeutic neurorehabilitation protocols in healthy participants or patients who suffer from changes in the CSE and brain activity level in some neurological disorders affecting the M1 and CB circuits, such as stroke or multiple sclerosis.

As this technique (concurrent bilateral a-tDCS_M1+CB_) may potentially modulate brain function dramatically, investigating its effects is important for using it as a treatment in patients with brain connectivity disorders, such as cerebellar ataxia or Parkinson's.

## Data Availability

Enquiries about data availability should be directed to the authors.

## Abbreviations

a-tDCS: Anodal transcranial direct current stimulation
a-tDCS_CB_: Bilateral a-tDCS of cerebellum
a-tDCS_M1_: Bilateral a-tDCS of M1
a-tDCS_Sham_: Bilateral sham a-tDCS
a-tDCS_M1+CB_: Concurrent bilateral a-tDCS of primary motor cortex and cerebellum
CB: Cerebellum
CSE: Corticospinal excitability
CNS: Central nervous system
DLPFC: Dorsolateral prefrontal cortex
EMG: Electromyography
FDI: First dorsal interosseous
ICF: Intracortical facilitation
LICI: Long intracortical inhibition
M1: Primary motor cortex
MEP: Motor evoked potential
PEST: Parameter estimation by sequential testing
RMT: Resting motor threshold
SICI: Short intracortical inhibition
tDCS: Transcranial direct current stimulation
TMS: Transcranial direct current stimulation
